# Prognostic impact of nutritional and inflammation-based risk scores in follicular lymphoma in the era of anti-CD20 targeted treatment strategies

**DOI:** 10.1007/s00432-021-03758-5

**Published:** 2021-08-20

**Authors:** Niklas Gebauer, Britta Mengler, Svenja Kopelke, Alex Frydrychowicz, Alexander Fürschke, Carsten Hackenbroch, Arthur Bauer, Armin Riecke, Nikolaus von Bubnoff, Sebastian Fetscher, Hanno M. Witte

**Affiliations:** 1 Department of Hematology and Oncology, University Hospital of Schleswig-Holstein, Campus Luebeck, Ratzeburger Allee 160, 23538 Lübeck, Germany; 2 Department of Radiology, University Hospital of Schleswig-Holstein, Campus Luebeck, 23538 Luebeck, Germany; 3 Department of Radiology, Federal Armed Forces Hospital Ulm, Oberer Eselsberg 40, 89081 Ulm, Germany; 4 Department of Hematology and Oncology, Federal Armed Hospital Ulm, Oberer Eselsberg 40, 89081 Ulm, Germany; 5 Department of Hematology and Oncology, Sana Hospitals Luebeck, Kronsforder Allee 71-73, 23560 Luebeck, Germany

**Keywords:** Risk scores, Inflammation, Follicular lymphoma, CRP, Albumin

## Abstract

**Background:**

The composition of the tumor microenvironment (TME) is conditioned by immunity and the inflammatory response. Nutritional and inflammation-based risk scores have emerged as relevant predictors of survival outcome across a variety of hematological malignancies.

**Methods:**

In this retrospective multicenter trial, we ascertained the prognostic impact of established nutritional and inflammation-based risk scores [Glasgow Prognostic Score (GPS), C-reactive–protein/albumin ratio (CAR), neutrophil–lymphocyte ratio (NLR), prognostic nutritional index (PNI), and prognostic index (PI)] in 209 eligible patients with histologically confirmed CD20^+^ follicular lymphoma (FL) of WHO grade 1 (37.3%), 1–2 (16.3%), 2 (26.8%) or 3A (19.8%) admitted to the participating centers between January 2000 and December 2019. Characteristics significantly associated with overall or progression-free survival (OS, PFS) upon univariate analysis were subsequently included in a Cox proportional hazard model.

**Results:**

In the study cohort, the median age was 63 (range 22–90 years). The median follow-up period covered 99 months. The GPS and the CAR were identified to predict survival in FL patients. The GPS was the only independent predictor of OS (*p* < 0.0001; HR 2.773; 95% CI 1.630–4.719) and PFS (*p* = 0.001; HR 1.995; 95% CI 1.352–2.944) upon multivariate analysis. Additionally, there was frequent occurrence of progression of disease within 24 months (POD24) in FL patients with a calculated GPS of 2.

**Conclusion:**

The current results indicate that the GPS predicts especially OS in FL patients. Moreover, GPS was found to display disease-specific effects in regard to FL progression. These findings and potential combinations with additional established prognosticators should be further validated within prospective clinical trials.

**Supplementary Information:**

The online version contains supplementary material available at 10.1007/s00432-021-03758-5.

## Background

Follicular lymphoma (FL) is a heterogeneous hematological neoplasm which is usually composed of follicle-center B cells (Pastore et al. 2015; Swerdlow et al. 2016). The current edition of the World Health Organization (WHO) classification categorizes FL as the most common subtype of indolent B-cell non-Hodgkin lymphoma (NHL) (Swerdlow et al. 2016). FL represents 20% of all lymphoma cases (Sant et al. 2010). It is more common in industrialized countries and shows an elevated incidence with increasing age (Anderson et al. 1998). The median age of onset is just over 65 years and the vast majority of patients present with advanced stage disease (Casulo 2016; Conconi et al. 2015). Women are affected more frequently than men (ratio 1.7: 1) (1997). Moreover, the WHO classification distinguishes different histological subtypes. FL is graded by estimating the absolute number of centroblasts in ten neoplastic follicles per microscopic high-power field (Nathwani et al. 1986). FL grade 3B is associated with the absence of centrocytes and is biologically more closely related to diffuse large B-cell lymphoma (DLBCL) than indolent FLs classified as grade 1–3A (Horn et al. 2011; Katzenberger et al. 2004).

Leading clinical feature in the majority of cases is a gradually progressive lymphadenopathy (Hiddemann and Cheson 2014). Approximately, 20% of FL cases present with progressive disease within the first 24 months after initial treatment (POD24) and these patients were shown to have an exceptionally bad outcome when treated with established immunochemotherapeutic regimens (Casulo et al. 2015). The Follicular Lymphoma International Prognostic Index (FLIPI) serves as a reliable predictor of survival outcomes in FL patients (Montoto et al. 2004; Solal-Celigny et al. 2004). However, the FLIPI is incapable of determining the convenient time for treatment initiation (Schans et al. 2009).

Treatment approaches are stage adapted and depend on comorbidities as well as performance status (PS) (Batlevi et al. 2020; Freedman 2018). Recently, alterations in *EZH2* gene locus could be identified as driver mutations harboring the capability to guide therapeutic decision making in advanced stage FL (Jurinovic et al. 2019; Szumera-CieCkiewicz et al. 2020). Comprehensive genomic profiling revealed distinct heterogeneity in FL with or without the genetic hallmark of FL represented by the chromosomal translocation t(14;18)(q32;q21), which leads to an overexpression of the BCL-2 protein (Kretzmer et al. 2015; Nann et al. 2020; Qu et al. 2019; Stevens et al. 2017).

As comprehensive genomic profiling is not performed on a routine basis, and clinical or laboratory predictors are required to individualize treatment options for FL patients, anticipating adverse outcomes including POD24.

There is growing evidence for the prognostic impact of the tumor microenvironment (TME) in cancer patients (Keane et al. 2017; Rieken et al. 2020). The cellular composition of the TME mirrors the degree of systemic inflammation. Several biomarkers such as the C-reactive protein (CRP) and albumin are able to reflect the inflammatory response in lymphoma patients. The activity of tumor-infiltrating immune cells and proinflammatory cytokines regulate serological CRP levels (Al Murri et al. 2006; He et al. 2018). Moreover, a nutritional component represents an essential element of different risk scores or ratios and staging systems (Tadmor et al. 2015). Frequently, this nutritional aspect is indicated by albumin serum level at initial diagnosis.

In previous studies, scores and ratios comprising metabolic and inflammatory parameters from the peripheral blood were established as independent predictors of clinical outcome in various malignancies such as colorectal cancer, multiple myeloma, Hodgkin lymphoma or DLBCL (Dolan et al. 2018a,b; Hao et al. 2017; Marcheselli et al. 2017; Reddy et al. 2018; Tadmor et al. 2015; Witte et al. 2019,2020). One such inflammation- and nutrition-based risk score is the Glasgow Prognostic Score (GPS) which differentiates three subgroups (group I: 0 points; group II: 1 point; group III: 2 points) by calculating one point for serum albumin of < 35 g/L and another point for CRP levels of > 10 mg/dl (Witte et al. 2019). Moreover, one of its variants the CRP/albumin ratio (CAR) constitutes another nutritional- and inflammation-based risk score.

In the current study, our aim was to evaluate different nutritional and inflammation-based risk scores in comprehensive multi-institutional cohort of FL patients as a complementary resource for risk stratification.

## Material and methods

In this retrospective multicenter trial, we investigated the prognostic impact of different scoring systems and ratios containing inflammatory and/or nutritional parameters or immune cells from the peripheral blood at initial diagnosis in FL patients within the context of optimal risk stratification. Patients with FL from the participating departments for Hematology and Oncology of the University Hospital Schleswig–Holstein (UKSH Campus Lübeck) and the Sana Hospital Lübeck that received cytoreductive treatment between January 2000 and December 2019 were screened with regard to their inclusion in the present study. Initial screening identified 285 FL patients. Patients with insufficient follow-up (19 patients referred to other institutions within 30 days after initial diagnosis and subsequent loss of follow-up in 8 patients) and patients where a watch and wait strategy was pursued (*n* = 40) were excluded. FL patients who experienced lymphoma progression after an initial watch and wait period would have been considered for the current study, if a cytoreductive treatment had been administered within the course of the disease. Additionally, patients with FL that transformed to DLBCL (tFL) or with an underlying histological grade 3B harboring an aggressive clinical course were excluded as well (*n* = 9). Moreover, FL patients with an underlying HIV infection were excluded. Entirely, 209 FL patients proceeded to further analyses.

### Clinicopathological characteristics

Clinical information was collected from the original electronic patient files. Data collection included staging data, the Eastern Cooperative Oncology Group performance status (ECOG-PS), the Charlson Comorbidity Index (CCI), treatment modalities, treatment responses and pattern of relapse (Charlson et al. 1987; Verger et al. 1992).

Laboratory data incorporated parameters from the baseline differential blood count, serum levels of lactate dehydrogenase (LDH), the beta-2-microglobulin (B2M) as well as the inflammation-related parameters CRP and albumin at primary diagnosis.

Centralized hematopathological review of formalin-fixed and paraffin-embedded (FFPE) tissue samples was performed in 143/209 (68.4%) cases. In those cases, initial diagnosis was confirmed by at least two independent pathologists (HM and ACF) in accordance with current WHO recommendations (Swerdlow et al. 2016).

### Prognostic scoring systems

On a routine clinical practice, the FLIPI as well as the modified FLIPI-2 was calculated for all FL patients based on the aforementioned clinical and laboratory characteristics (Numata et al. 2012).

For the evaluation of inflammatory-based risk scores and ratios, we calculated established prognostic scores that incorporate immune cells such as neutrophils and lymphocytes. These included the widely accepted neutrophil-to-lymphocyte ratio (NLR) (Azab et al. 2013; Azuma et al. 2019; Dolan and McMillan 2017; He et al. 2013; Reddy et al. 2018). The prognostic nutritional index (PNI) additively takes into account the patient’s nutritional status in the form of albumin (PNI = albumin + 0.005 × total lymphocyte count) and the prognostic index (PI) regards the acute phase protein CRP (> 10 mg/dl) as well as the total white blood cell count (> 10 × 10^9^/L) accounting one point for each feature (Dolan et al. 2018b; Kasymjanova et al. 2010). As introductorily illustrated, we have calculated the GPS connoting one point for a CRP value > 10 mg/dl and another point for serum albumin < 35 g/L. As a result, the GPS distinguishes three subgroups (group I: 0 points; group II: 1 point; group III: 2 points) (Hao et al. 2017). Moreover, we calculated the ratio of CRP and albumin (CAR) for each case. All risk scores and ratios gathered in the present study are depicted in Table 1.

**Table 1:** Systemic inflammation-based prognostic ratios and scores

Ratio/score	Ratio/score
NLR	
Neutrophil count:lymphocyte count	≤ 3
Neutrophil count:lymphocyte count	3–5
Neutrophil count:lymphocyte count	> 5
PI	
White blood cell count ≤ 10 × 10^9^/l and C-reactive protein ≤ 10 mg/l	0
White blood cell count ≤ 10 × 10^9^/l and C-reactive protein > 10 mg/l	1
White blood cell count > 10 × 10^9^/l and C-reactive protein ≤ 10 mg/l	1
White blood cell count > 10 × 10^9^/l and C-reactive protein > 10 mg/l	2
PNI	
Albumin (g/l) + 5 × (lymphocyte count (10^9^/l))	≤ 50
Albumin (g/l) + 5 × (lymphocyte count (10^9^/l))	> 50
CAR	
C-reactive protein/albumin	≤ 0.22
C-reactive protein/albumin	> 0.22
GPS	
C-reactive protein ≤ 10 mg/l and albumin ≥ 35 g/l	0
C-reactive protein > 10 mg/l or albumin < 35 g/l	1
C-reactive protein > 10 mg/l and albumin < 35 g/l	2

*NLR* neutrophil–lymphocyte ratio, *PI* prognostic index, *PNI* prognostic nutritional index, *CAR* C-reactive-protein/albumin ratio, *GPS* Glasgow Prognostic Score

### Treatment and responses

Staging was performed according to the Cotswold modifications of the Ann Arbor classification. Open lymph node excision or needle core biopsies of suspect lymph nodes were performed at initial diagnosis. Afterward, a stage-adapted decision regarding the administration of reductive agents or radiation therapy was made on the basis of the treating physician’s choice with current GLSG (German Lymphoma Study Group)/GLA (German Lymphoma Alliance) study protocols serving as an institutional standard where applicable. Treatment response was defined in keeping with the established International Workshop Criteria (IWSC) of complete remission (CR) and partial remission (PR) (Brepoels et al. 2007). Fluorodeoxyglucose positron emission tomography (FDG-PET) scans were not performed on a routine basis. Overall survival (OS) and progression-free survival (PFS) were calculated from the date of initial diagnosis. The assessment of the treatment-related toxicity profile was conducted in conformity with the National Cancer Institute Common Toxicity (NCI CTC; version 2.0) (Kaba et al. 2004).

### Ethics statement

This retrospective study was approved by the ethics committee of the University of Luebeck (Reference-no 18-037) and conducted in accordance with the Declaration of Helsinki. Written informed consent referred to routine diagnostics and academic assessment of the archived biopsy specimen as well as transfer of clinical data was obtained from all patients.

### Statistics

All statistical analyses were conducted using GraphPad PRISM6 (GraphPad Software Inc., San Diego, CA, USA), SPSS 26 (IBM, Armonk, NY, USA) and R v4.0.2. Kolmogorov–Smirnov test was performed to assess normality of distribution. Initially, cutoff values for NLR, CAR, and PNI were selected from previously published data investigating the prognostic impact of nutritional and inflammation-based risk scores/ratios in cancer patients (Dolan et al. 2018b; Witte et al. 2020). Consecutively, cutoff value confirmation has been performed utilizing a receiver operating characteristic (ROC) analysis proposed by Budczies et al. (2012). Survival (OS and PFS) was calculated by means of the Kaplan–Meier method. Subsequently, we performed a confirmatory univariate Cox analysis. Significance level was defined at *p* < 0.05. A subsequent multivariate proportional hazard model was conducted for characteristics exhibiting a trend toward statistical significance (*p* < 0.07) that were found to be associated with OS or PFS within both univariate approaches. The Mann–Whitney *U* test and the Chi-squared test were used to assess differences between subgroups of FL patients, as appropriate. In anticipation of non-linear relationships between variables, the Pearson’s correlation analysis was applied. Comparative analysis regarding the prognostic impact of the GPS and the CAR was performed by estimating the Harrel’s concordance index (c-index) and the corrected Akaike’s information criterion (cAIC) to identify the most qualified CRP and albumin-based risk score (Supplementary Methods) (Akaike 1974; Heller and Mo 2016).

## Results

### Clinical characteristics

Clinicopathological characteristics are briefly depicted in Table 2. Gender distribution was balanced within the study cohort (male 51.7%/ female 48.3%). The median age at diagnosis was 63 years (range 22–90 years) and the median follow-up was 99 months (3–397 months, 25% percentile 62.0; 75% percentile 154.0). The median body mass index (BMI) was 25.38 kg/m^2^, ranging from 17.8 kg/m^2^ to 46.71 kg/m^2^. An elevated ECOG-PS of ≥ 2 was present in only 27 patients (12.9%). The higher the GPS, the more FL patients presented with B symptoms (GPS 0 = 14.4%; GPS 1 = 17.1% and GPS 2 = 27.8%). Histological grading was found to be equally distributed among various GPS groups. The distribution of composite scores/ratios and their component values is outlined in Table 3. Considering the nutritional- and inflammation-based risk scores/ratios, only the minority of FL patients presented with features of systemic inflammation at initial diagnosis [NLR > 5 (27.7%); CAR > 0.22 (28.7%); GPS = 2 (17.2%); PNI < 50 (36.4%); PI = 2 (4.3%)]. Table 4 illustrates relationships between blood cell-based as well as nutritional- and inflammation-based risk scores/ratios and essential clinical features of FL patients. A large fraction of calculated scores/ratios correlated significantly with age, ECOG-PS, elevated LDH levels, the CCI and the FLIPI.

**Table 2:** Baseline clincopathological characteristics in the current study cohort

GPS	Overall study group (*n* = 209)	Group I GPS 0 (*n* = 132)	Group II GPS 1 (*n* = 41)	Group III GPS 2 (*n* = 36)
Male/female	108 (51.7%)/101 (48.3%)	63 (47.7%)/69 (52.3%)	22 (53.7%)/19 (46.3%)	23 (63.9%)/13 (36.1%)
Median age (range), years	63 (22–90)	60 (22–90)	65 (44–82)	67.5 (45–88)
BMI (median, range)	25.3 (17.8–46.7)	25.5 (18.1–46.7)	25.4 (17.8–43.6)	24.4 (18.7 -30.7)
Weight disorder				
Cachexia (BMI < 20 kg/m^2^)	17 (8.1%)	9 (6.8%)	4 (9.8%)	4 (11.1%)
Obesity (BMI > 30 kg/m^2^)	29 (13.9%)	19 (14.4%)	9 (21.9%)	1 (2.8%)
ECOG PS				
0–1	182 (87.1%)	123 (93.2%)	35 (85.4%)	24 (66.7%)
2–4	27 (12.9%)	9 (6.8%)	6 (14.6%)	12 (33.3%)
CCI (median, range)	4 (0–10)	4 (0–8)	4 (2–9)	4 (2–10)
Extranodal sites				
0–1	163 (77.9%)	106 (80.3%)	32 (78.0%)	25 (69.4%)
≥ 2	46 (22.1%)	26 (19.7%)	9 (22.0%)	11 (30.6%)
LDH level				
< 240 U/l	157 (75.1%)	116 (87.9%)	24 (58.5%)	17 (47.2%)
> 240 U/l	52 (24.9%)	16 (12.1%)	17 (41.5%)	19 (52.8%)
Albumin (g/l) (median, range)	40.9 (13.9–51.2)	42.1 (34.0–51.2)	37.0 (25.0–50.4)	31.7 (13.9–34.5)
≥ 35 g/l	152 (72.7%)	131 (99.2%)	21 (51.2%)	–
< 35 g/l	57 (27.3%)	1 (0.8%)	20 (48.8%)	36 (100.0%)
CRP (mg/dl) (median, range)	3.5 (0.0–297.0)	1.8 (0.0–9.3)	10.1 (0.1–159.9)	16.2 (11.6–297.0)
≤ 10 mg/dl	152 (72.7%)	132 (100.0%)	20 (48.8%)	–
> 10 mg/dl	57 (27.3%)	–	21 (51.2%)	36 (100.0%)
Histological grading				
1	78 (37.3%)	59 (44.7%)	7 (17.1%)	12 (33.3%)
1–2	34 (16.3%)	19 (14.4%)	11 (26.8%)	4 (11.1%)
2	56 (26.8%)	31 (23.5%)	15 (36.6%)	10 (27.8%)
3A	41 (19.6%)	23 (17.4%)	8 (19.5%)	10 (27.8%)
Ann Arbor stage				
I	26 (12.4%)	17 (12.9%)	5 (12.2%)	4 (11.1%)
II	32 (15.3%)	21 (15.9%)	9 (22.0%)	2 (5.6%)
III	60 (28.7%)	39 (29.5%)	11 (26.8%)	10 (27.8%)
IV	91 (43.5%)	55 (41.7%)	16 (39.0%)	20 (55.5%)
FLIPI				
Median (range)	2 (0–5)	2 (0–4)	2 (0–5)	3 (0–5)
Low risk 0–1	68 (32.5%)	50 (37.9%)	9 (22.0%)	9 (25.0%)
Intermediate risk 2	68 (32.5%)	47 (35.6%)	13 (31.7%)	8 (22.2%)
High risk ≥ 3	73 (35.0%)	35 (26.5%)	19 (46.3%)	19 (52.8%)
FLIPI-2				
Median (range)	1 (0–5)	1 (0–5)	2 (0–4)	2 (0–4)
Low risk 0	35 (16.8%)	30 (22.7%)	2 (4.9%)	3 (8.3%)
Intermediate risk 1–2	138 (66.0%)	87 (65.9%)	30 (73.1%)	21 (58.4%)
High risk ≥ 3	36 (17.2%)	15 (11.4%)	9 (22.0%)	12 (33.3%)

*BMI* body mass index, *CCI* Charlson Comorbidity Index, *CRP* C-reactive protein, *ECOG PS* Eastern Cooperative Oncology Group performance status, *FLIPI* Follicular Lymphoma International Prognostic Index, *GPS* Glasgow Prognostic Score, *LDH* lactate dehydrogenase

**Table 3:** The relationship between composite ratios and cumulative scores and their component values in FL patients (*n* = 209)

	*n* (%)	Median (range)	Median (range)
		Neutrophil (× 10^9^/l)	Lymphocyte (× 10^9^/l)
NLR			
≤ 3	98 (46.9%)	3.68 (0.39–9.83)	1.94 (0.38–36.18)
3–5	53 (25.4%)	4.32 (1.40–7.94)	1.19 (0.40–2.18)
> 5	58 (27.7%)	5.42 (1.52–21.83)	1.50 (0.22–5.45)

		Albumin (g/l)	CRP (mg/dl)
CAR			
≤ 0.22	149 (71.3%)	41.9 (25.0–51.2)	1.9 (0.0–10.3)
> 0.22	60 (28.7%)	32.9 (13.9–50.4)	18.3 (7.9–297.0)
GPS			
0	132 (63.2%)	42.1 (34.0–51.2)	1.9 (0.0–9.3)
1	41 (19.6%)	35.6 (25.0–50.4)	9.9 (0.1–159.9)
2	36 (17.2%)	31.7 (13.9–34.5)	16.7 (11.6–297.0)

		Albumin (g/l)	Lymphocyte (× 10^9^/l)
PNI			
≥ 50	133 (63.6%)	42.6 (30.4–51.2)	1.62 (0.24–36.18)
< 50	76 (36.4%)	33.0 (13.9–42.7)	0.74 (0.22–2.84)

		WBC (× 10^9^/l)	CRP (mg/dl)
PI			
0	134 (64.1%)	5.52 (1.60–8.93)	2.0 (0.0–9.3)
1	66 (31.6%)	6.19 (0.78–15.31)	14.8 (0.2–297.0)
2	9 (4.3%)	11.49 (11.00–45.36)	19.5 (10.3–137.0)

*NLR* neutrophil–lymphocyte ratio, *CAR* C-reactive protein albumin ratio, *CRP* C-reactive protein, *GPS* Glasgow Prognostic Score, *PI* prognostic index, *PNI* prognostic nutritional index, *WBC* white blood cell count

**Table 4:** Pearson’s correlation between composite ratios and cumulative scores and baseline clinicopathological characteristics of patients with follicular lymphoma (*n* = 209)

	Age	Sex	B symptoms	ECOG	LDH	CCI	FLIPI	AA
NLR	0.448	0.633	0.263	0.262	**0.002**	0.479	0.093	0.468
PI	0.078	0.768	0.129	** < 0.0001**	** < 0.0001**	**0.041**	**0.009**	0.214
PNI	0.170	**0.001**	0.124	**0.019**	**0.003**	0.102	** < 0.0001**	0.834
GPS	**0.003**	0.057	0.075	** < 0.0001**	** < 0.0001**	**0.002**	**0.003**	0.332
CAR	**0.025**	0.195	0.130	** < 0.0001**	** < 0.0001**	**0.014**	0.083	0.933
Albumin	**0.001**	0.102	0.094	**0.001**	** < 0.0001**	**0.001**	**0.004**	0.200
CRP	**0.025**	0.437	0.065	**0.003**	** < 0.0001**	**0.010**	0.251	0.826

*AA* Ann Arbor stage, *BMI* body mass index, *CAR* C-reactive protein albumin ratio, *CCI* Charlson Comorbidity Index, *CRP* C-reactive protein, *ECOG* Eastern Cooperative Oncology Group, *FLIPI* Follicular Lymphoma International Prognostic Index, *GPS* Glasgow Prognostic Score, *LDH* lactate dehydrogenase, *NLR* neutrophil–lymphocyte ratio, *PI* prognostic index

*p < 0.05 is considered significant

### Treatment modalities

GELF (Groupe d’Etude des Lymphomes Folliculaires) criteria are commonly used to indicate cytoreductive treatment initiation in FL patients. Table 5 depicts the composition of GELF criteria in the current study cohort. Most common GELF criteria were B symptoms (17.2%) and the involvement of ≥ 3 lymph nodes extending over 3 cm (16.7%). The higher the GPS, the more were GELF criteria detected in FL patients. Exceptionally, the presence of a compression syndrome was more prevalent in patients with lower GPS. Frequently cytoreductive treatment was initiated independently from the presence of GELF criteria (n = 107; 51.2%), whereas a watch and wait strategy was preferred in only 46 cases (22.0%). In total, an anti-CD20 targeted therapeutic agent was given in 162 of 209 cases (77.5%).

**Table 5:** GELF criteria to indicate treatment initiation

GELF criteria	Overall study group (*n* = 209)	Group I GPS 0 (*n* = 132)	Group II GPS 1 (*n* = 41)	Group III GPS 2 (*n* = 36)
GELF criteria (average, range)	0.74 (0–6)	0.73 (0–6)	0.68 (0–4)	0.83 (0–4)
Bulk > 7 cm	11 (5.3%)	9 (6.8%)	1 (2.4%)	1 (2.8%)
≥ 3 sites, > 3 cm	35 (16.7%)	21 (15.9%)	7 (17.1%)	7 (19.4%)
B symptoms	36 (17.2%)	19 (14.4%)	7 (17.1%)	10 (27.8%)
Splenic enlargement*	9 (4.3%)	6 (4.5%)	1 (2.4%)	2 (5.6%)
Compression syndrome**	25 (12.0%)	19 (14.4%)	4 (9.6%)	2 (5.6%)
Serous effusion***	14 (6.7%)	8 (6.1%)	2 (4.9%)	4 (11.1%)
Leukemic phase****	16 (7.7%)	8 (6.1%)	4 (9.6%)	4 (11.1%)
Cytopenia*****	14 (6.7%)	9 (6.8%)	2 (4.9%)	3 (8.3%)

*GELF* Groupe d’Etude des Lymphomes Folliculaires, *GPS* Glasgow Prognostic Score

*With inferior margin below the umbilical line

**Ureteral, orbital, gastrointestinal

***Pleural or peritoneal irrespective of cell content

**** > 5.0 × 109/L circulating malignant cells

*****Granulocyte count < 1.0 × 10^9^/L and/or platelets < 100 × 10^9^/L

Treatment modalities, response rates and associated toxicity profile are outlined in Table 6. Further treatment information for relapse setting is briefly summarized in Supplementary Tables 1 and 2 and the Supplementary Material.

**Table 6:** First-line treatment modalities of all FL patients included in the study

Characteristics	Overall study group (*n* = 209)	GPS 0 (*n* = 132)	GPS 1 (*n* = 41)	GPS 2 (*n* = 36)
Watch and wait	46 (22.0%)	38 (28.8%)	4 (9.8%)	4 (11.1%)
1st line treatment				
CHOP*-*like	89 (42.6%)	52 (39.4%)	20 (48.8%)	17 (47.2%)
Bendamustine	56 (26.8%)	41 (31.1%)	7 (17.1%)	8 (22.2%)
R based	130 (62.2%)	79 (59.8%)	27 (65.9%)	24 (66.7%)
O based	32 (16.7%)	14 (10.6%)	10 (24.4%)	8 (22.2%)
Radiation therapy	46 (22.0%)	31 (23.5%)	8 (19.5%)	7 (19.4%)
Other	18 (8.6%)	9 (6.8%)	5 (12.2%)	4 (11.1%)
Refusal	2 (1.0%)	–	–	2 (5.6%)
Anti-CD20 maintenance	90 (43.1%)	58 (43.9%)	18 (43.9%)	14 (38.9%)
Best response (IWSC)				
CR	82 (39.2%)	53 (40.2%)	16 (39.0%)	13 (36.1%)
PR	92 (44.0%)	57 (43.2%)	20 (48.8%)	15 (41.7%)
SD	24 (11.5%)	17 (12.9%)	4 (9.8%)	3 (8.3%)
PD	11 (5.3%)	5 (3.8%)	1 (2.4%)	5 (13.9%)
POD24	37 (17.7%)	14 (10.6%)	8 (19.5%)	15 (41.7%)
Dfd	38 (18.2%)	4 (3.0%)	4 (9.8%)	30 (83.3%)
Toxicity profile (NCI CTC)				
Cytopenia grade III/IV	34 (16.3%)	20 (15.2%)	8 (19.5%)	6 (16.7%)
Neutropenia-related fever	15 (7.2%)	7 (5.3%)	4 (9.8%)	4 (11.1%)
Pneumonia	6 (2.9%)	3 (2.3%)	1 (2.4%)	2 (5.6%)
Sepsis	8 (3.8%)	4 (3.0%)	2 (4.9%)	2 (5.6%)
Neuropathy	19 (9.1%)	12 (9.1%)	4 (9.8%)	3 (8.3%)
Cardiotoxicity	8 (3.8%)	3 (2.3%)	2 (4.9%)	3 (8.3%)
Mucositis	11 (5.3%)	7 (5.3%)	3 (7.3%)	1 (2.8%)
Hematopoietic stem cell transplantation (HSCT) required in relapse/refractory disease				
Autologous HSCT	23 (11.0%)	10 (10.6%)	6 (9.8%)	7 (19.4%)
Allogenic HSCT	2 (1.0%)	2 (1.5%)	-	-

*CHOP* cyclophosphamide/hydroxydaunorubicin/vincristine/prednisolone, *CR* complete remission, *Dfd* death from disease, *GPS* Glasgow Prognostic Score, *HSCT* hematopoietic stem cell transplantation, *IWSC* International Workshop criteria, *NCI CTC* National Cancer Institute Common Toxicity Criteria, *O* obinutuzumab, *PD* progressive disease, *POD24* progression of disease within 24 months, *PR* partial remission, *R* rituximab, *SD* stable disease

### Clinical risk stratification

The majority of patients presented with advanced stage disease (Ann Arbor stage III/IV 151/209; 72.2%) at initial diagnosis. This fact was found to be equally distributed among the various GPS subgroups. However, the higher the GPS, the higher is the FLIPI as well as the FLIPI-2. Pearson’s correlation analysis revealed the close connection between systemic inflammation reflected by the GPS and increasing FLIPI (*p* = 0.003) as demonstrated in Table 4. Moreover, correlation analysis showed strong relations between the PNI and sex as well as the GPS and age. Therefore, we performed separate analyses to specify the value of nutritional- and inflammation-based risk scores in different age and sex subgroups. Age-related survival analysis revealed the GPS to significantly predict OS independent of age, while the GPS predicts PFS only in FL patients with advanced age (> 60 years), but not in FL patients < 60 years. (Supplementary Figure 1). Irrespective of sex subgroups the PNI hold its prognostic value concerning OS, but not PFS (Supplementary Figure 1). Hence, prognostic implications of the GPS and the PNI sustained additional age-related and sex-related analysis, respectively. There was extranodal disease in 46 (22.1%) FL patients. Upon univariate as well as subsequent multivariate analysis, the FLIPI maintained significant impact on OS (*p* = 0.047), but not PFS (*p* = 0.109) (Tables 7 and 8, Supplementary Figure 2).

**Table 7:** Progression-free and overall survival in univariate analysis (univariate Cox analysis)

Prognostic factor	PFS		OS	
	*p* value	HR (95% CI)	*p* value	HR (95% CI)
Univariate analysis				
GPS	** < 0.0001**	1.755 (1.387–2.221)	** < 0.0001**	3.334 (2.432–4.569)
CRP	0.304	1.231 (0.828–1.830)	** < 0.0001**	6.502 (3.419–12.367)
Albumin	** < 0.0001**	2.524 (1.680–3.793)	** < 0.0001**	7.184 (4.057–12.724)
NLR	0.062	1.381 (0.984–1.938)	0.595	1.169 (0.657–2.079)
CAR	**0.001**	1.999 (1.330–3.003)	** < 0.0001**	6.561 (3.717–11.579)
PI	**0.007**	1.540 (1.127–2.104)	** < 0.0001**	3.182 (2.124–4.766)
PNI	0.075	0.679 (0.444–1.040)	** < 0.0001**	0.253 (0.119–0.536)
Age > 60 years	0.544	0.884 (0.594–1.316)	** < 0.0001**	4.040 (2.049–7.963)
B symptoms	0.053	1.600 (0.994–2.575)	**0.035**	1.898 (1.045–3.445)
ECOG PS ≥ 2	0.086	1.599 (0.935–2.733)	** < 0.0001**	3.509 (1.871–6.581)
Elevated LDH	0.058	1.521 (0.987–2.344)	** < 0.0001**	3.863 (2.241–6.658)
BM involvement	**0.002**	1.869 (1.247–2.800)	0.277	1.364 (0.780–2.387)
CCI > 3	0.544	0.884 (0.593–1.317)	** < 0.0001**	3.773 (1.932–7.367)
FLIPI	0.090	1.236 (0.967–1.580)	**0.001**	1.804 (1.269–2.565)
Ann Arbor	**0.009**	2.006 (1.189–3.384)	0.444	1.285 (0.676–2.445)

Bold values indicate statistical significance (*p* < 0·05) in univariate cox analysis

*CAR* C-reactive protein–albumin ratio, *CCI* Charlson Comorbidity Index, *CRP* C-reactive protein, *ECOG PS* Eastern Cooperative Oncology Group performance status, *FLIPI* Follicular Lymphoma International Prognostic Index, *GPS* Glasgow Prognostic Score, *HR* hazard ratio, *LDH* lactate dehydrogenase, *NLR* neutrophil-to-lymphocyte ratio, *OS* overall survival, *PFS* progression-free survival, *PI* prognostic index

**Table 8:** Overall survival and progression-free survival in univariate analysis and consecutive multivariate Cox proportional hazard regression

Prognostic factor	Univariate analysis OS	Multivariate analysis OS	
	*p* value	*p* value	HR (95% CI)
GPS	** < 0.0001**	** < 0.0001**	2.773 (1.630–4.719)
PI*	** < 0.0001**	0.808	1.093 (0.534–2.238)
PNI**	** < 0.0001**	0.455	0.717 (0.301–1.713)
CCI > 3	** < 0.0001**	0.065	1.942 (0.961–3.926)
FLIPI	**0.001**	**0.047**	1.454 (1.005–2.103)

	Univariate analysis PFS	Multivariate analysis PFS	
	*p* value	*p* value	HR (95% CI)
GPS	** < 0.0001**	**0.001**	1.995 (1.352–2.944)
PI*	**0.007**	0.394	0.800 (0.478–1.337)
PNI**	0.075	0.889	1.036 (0.632–1.698)
CCI > 3	0.544	0.109	0.712 (0.469–1.079)
FLIPI	0.090	0.158	1.206 (0.930–1.565)

*CCI* Charlson Comorbidity Index, *FLIPI* Follicular Lymphoma International Prognostic Index, *GPS* Glasgow Prognostic Score, *OS* overall survival, *PFS* progression-free survival, *PI* prognostic index, *PNI* prognostic nutritional index

*CRP > 10 mg/dl, white blood cell count > 11,000/μl

** > 50

### Prognostic scoring systems

Univariate Cox analysis revealed potential prognostic impact of assessed scores/ratios on PFS and OS (Table 7). Especially, the GPS, the CAR and the PI were found to predict PFS and OS upon univariate analysis. In concurrence with results from previous studies on hematological malignancies, univariate analysis revealed CRP and albumin as individual components of the GPS and CAR to have significant impact on OS (*p* < 0.0001; *p* < 0.0001) (Witte et al. 2020). Albumin exhibited significant impact only on PFS (*p* < 0.0001).

Characteristics or scores/ratios significantly associated with PFS or OS upon univariate analysis underwent subsequent confirmatory multivariate analysis (Table 8). Upon model analysis calculating the c-index and the cAIC, the GPS revealed predictive superiority for both OS and PFS compared to CAR. This analysis has been performed to select the superior CRP/albumin-based score for further comparative multivariate analysis (Supplementary Table 3). Comparative multivariate analysis confirmed prognostic implications of the GPS to be the only independent predictor of PFS (*p* = 0.001; HR 1.995; 95% CI 1.352–2.944) as well as OS (*p* < 0.0001; HR 2.773; 95% CI 1.630–4,719). The FLIPI was found to predict OS (*p* = 0.047; HR 1.454; 95% CI 1.005–2.103), but not PFS (*p* = 0.158; HR 1.206; 95% CI 0.930–1.565) upon multivariate analysis. While the dichotomization of the PI, the PNI and the CCI > 3 in the univariate analysis revealed to significantly predict OS or PFS, but these results could not be confirmed in subsequent multivariate analysis.

Moreover, due to the fact that cachexia (*n* = 17; BMI < 20 kg/m^2^) and obesity (n = 29; BMI > 30 kg/m^2^) display relevant risk factors in cancer patients, we additionally performed univariate as well as subsequent multivariate analyses after the exclusion of those patients to reduce confounding in the present cohort. Notwithstanding the exclusion of FL patients with weight disorders, multivariate analysis revealed comparable results regarding the identification of independent risk factors. Therefore, the GPS has been identified as the only independent risk factor for both PFS and OS (Supplementary Tables 4 and 5).

**Fig. 1:**
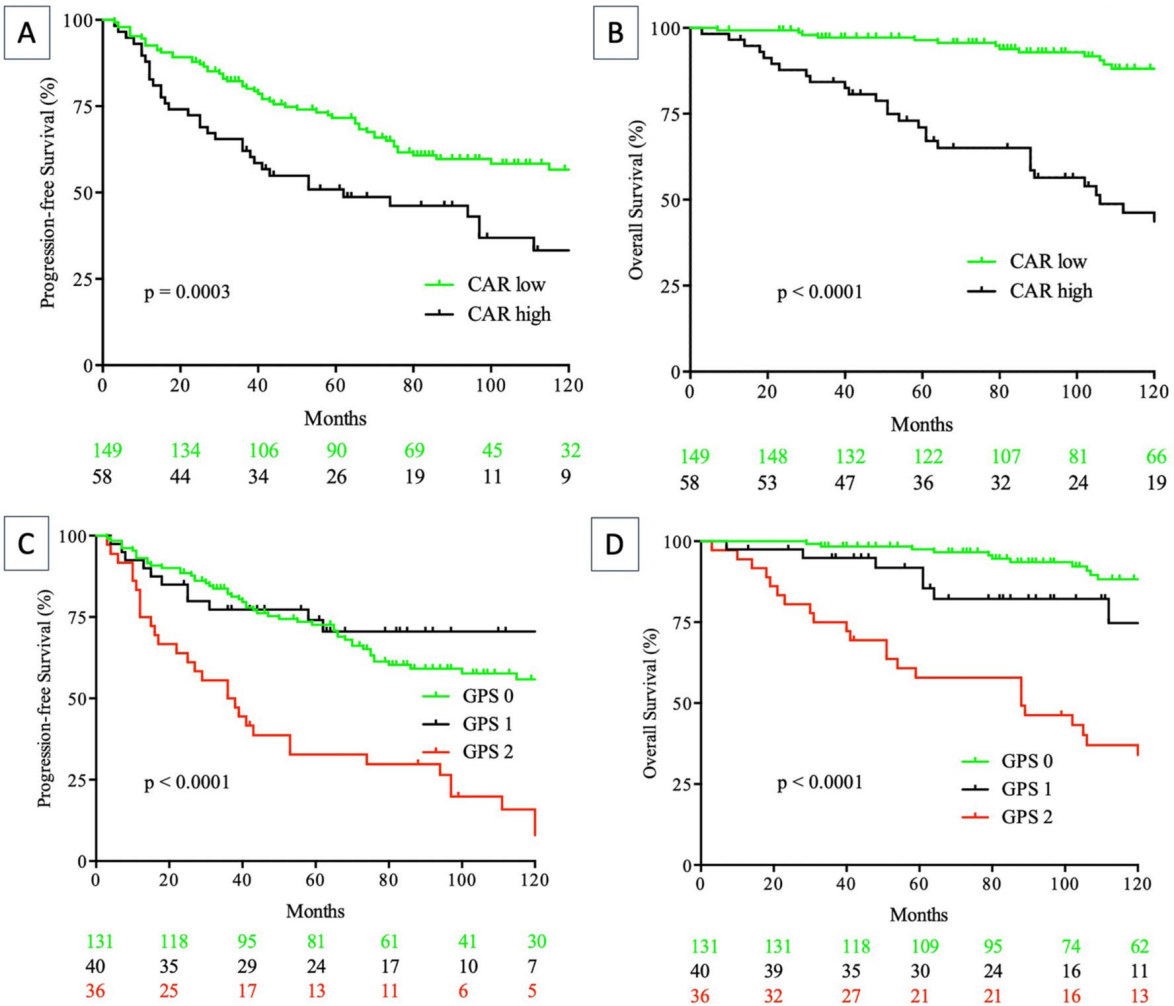
Progression-free (**A**, **C**) and overall (**B**, **D**) survival according to CRP/albumin ratio (CAR) (log-rank test; **A**, **B**) and Glasgow Prognostic Score (GPS) (log-rank GPS 0 vs. GPS 1 vs. GPS 2; **C**, **D**) in follicular lymphoma patients

Kaplan–Meier analysis visualizes the influence of the GPS and the CAR on PFS (*p* < 0.0001; *p* = 0.0003) and OS (*p* < 0.0001; *p* < 0.0001) (Fig. 1).

In the light of a median follow-up of 99 months, median PFS among the GPS subgroups was 75 months (GPS 0), 63 months (GPS 1) and 37 months (GPS 2), respectively. Moreover, 5-year PFS was 64.1% for the GPS 0 subgroup, 57.5% for the GPS 1 subgroup and 30.6% for FL patients with a GPS of 2. During the follow-up period of 99 months in median, 54 lymphoma-related deaths were recorded (25.8%) and 102 FL patients (48.8%) experienced relapse event or refractory disease. Interestingly, 36 FL patients presented with POD24 (17.7%) within the study cohort, of which 15 were categorized with a GPS of 2 (15/36 cases; 41.7%), while 17 patients (47.2%) presented with a PNI ≥ 45 and only two patients had a PI of 2 (5.6%). In this subgroup of FL patients presenting with POD24, an NLR ≥ 5 has been calculated in 11 cases.

## Discussion

The current study is the first to evaluate the prognostic capabilities of nutritional- and inflammatory-based risk scores/ratios in follicular lymphoma patients in the era of anti-CD20 directed treatment strategies. Previous studies have proven the potential prognostic impact across various solid malignancies such as non-small cell lung cancer, biliary tract cancer and colorectal carcinoma (Dolan et al. 2018b; Moriwaki et al. 2014; Shiba et al. 2015; Yotsukura et al. 2016). Recently, we provided evidence for the GPS serving as a complementary resource for risk stratification in several hematological neoplasms harboring the potential to predict both PFS and OS (Witte et al. 2019,2020). In comparison to other scores and ratios, several studies have demonstrated the prognostic superiority of the GPS (Dolan et al. 2018a; Hao et al. 2017). The pathophysiological background of the GPS is that the intensity of systemic inflammation and decreasing nutritional status elicited by unspecific immune response and the consumptive character of an underlying malignant disease has significant impact on progression of disease (Proctor et al. 2011). Systemic inflammation is reflected by the measurement of the CRP level in serum, while the nutritional aspect can be projected on albumin levels (Al Murri et al. 2006). Both measurements should be determined at initial diagnosis. Although Dolan et al*.* provided evidence for the superiority of inflammation-based scores for risk stratification in contrast with inflammation-derived ratios of peripheral blood, the CAR, which comprises the same components as the GPS, was found to significantly predict PFS as well as OS upon univariate analysis (Dolan et al. 2017, 2018a). Due to redundance of the individual components and the expected loss of independency of the GPS upon further multivariate analysis, we previously compared both CRP and albumin-based risk scores (GPS and CAR) by calculating the c-index and the cAIC. In this context, we identified the GPS to hold more accurate prognostic capabilities. Therefore, the CAR was not included in consecutive multivariate analysis.

The comparative analysis of different scores and ratios which are based on markers derived from white blood cell count reflecting acute phase reaction or the patients’ myeloid and/or lymphoid response revealed that there is exclusive prognostic impact on survival for CRP and albumin-based scoring systems. Moreover, established scoring systems based on cellular components from the peripheral blood such as the NLR were not found to significantly predict PFS or OS. Additionally, the PI or the PNI comprising CRP or albumin for calculation were not able to hold statistical significance in regard to its prognostic value upon multivariate analysis, either. Notably, the majority of the considered scores or ratios have been developed and validated for solid cancer entities (Azab et al. 2013; He et al. 2013; Liu et al. 2016; Shimada et al. 2010). The importance of nutritional as well as inflammatory aspects has been positively evaluated for several hematological malignancies and we now expand this notion into prognostication for FL.

We found close and significant correlations between the GPS and individual components (age, elevated LDH levels) of the FLIPI, the FLIPI itself, the ECOG performance status and the CCI (Table 4). In concurrence with previously published data, the FLIPI was identified to have significant prognostic impact on OS (Solal-Celigny et al. 2004).

However, in contrast to previously published studies, the GPS did not separate survival curves adequately in low-risk patients, while there was no significant distinction between FL patients with a calculated GPS of 0 compared to those with a calculated GPS of 1 (Fig. 1). Therefore, FL patients had to suffer from both a systemic inflammatory component and a consumptive nutritional component (GPS 2) to be allocated into a clinically adverse risk group. This factor might serve as an indicator for the missing prognostic value of several scoring systems other than the GPS or the CAR.

Another merit of the GPS is that the score can be calculated very quickly and easily on the basis of routine laboratory parameters. Therefore, the calculation of the GPS is an extremely cost-effective and readily available tool to predict survival in cancer patients. Further, more effortful diagnostics such as cytogenetic or molecular analysis are associated with a certain latency, whereas the GPS can be determined immediately. There is also the fact that biomarkers that have been established for risk stratification in follicular lymphomas are less representative to display systemic inflammatory features. Several previous studies analyzing the prognostic value of the GPS demonstrated its robustness regarding the predictive efficiency in hematological malignancies (Hao et al. 2017; Li et al. 2013). The present data seem to confirm these previous findings across different age groups independent of potential confounders such as cachexia or obesity and expand the GPS-related prognostic spectrum by follicular lymphoma.

An important marker for adverse clinical outcomes in FL patients is the evaluation of POD24 (Casulo 2016; Casulo et al. 2015). Several studies aimed to predict POD24 by analyzing different biomarkers and genomic signatures. One such genetic biomarker that holds the potential to significantly influence therapeutic responses is an underlying *EZH2* mutation (Szumera-CieCkiewicz et al. 2020). Apart from that, a distinct genomic signature for the prediction of POD24 remains undetected. In the current study, we found an association between an elevated GPS and POD24. Accordingly, 41.7% of FL patients with a GPS of 2 developed progression of disease within 24 months (POD24 in 15/36 cases). Consequently, given the independency upon multivariate analysis, the GPS could serve as an excellent contributor for the identification of FL patients at risk.

Potential shortcomings of the present study include its limited sample size and its retrospective design harboring the perpetual eventuality of fragmentary data alongside heterogeneous treatment approaches. Although we were able to evaluate the causes of death in the majority of cases, the cause of death remains unknown for a subset of patients due to insufficient follow-up. Concurrent infections at initial diagnosis harbor the potential to distort the calculation of the GPS. Hence, FL patients considered for the inclusion in the study were screened for infections that possibly bias scoring results. A period of 30 days after initial clinical presentation was acknowledged to determine an alternative date for another blood sampling to exclude any relevant infection affecting the calculation of the GPS. Another essential limitation of the present study is lack of comprehensive cytogenetic analyses. Therefore, the presence or the absence of an underlying translocation t(14;18)(q32;q21) which displays the genetic hallmark of follicular lymphomas or further chromosomal alterations could not be considered in terms of prognostic capabilities in all cases and was therefore omitted from subsequent analysis (Alig et al. 2019; Qu et al. 2019). Moreover, due to the lack of analyzing molecular aberrations, the calculation of the recently established m7-FLIPI remained undeterminable (Jurinovic et al. 2019).

Current results demonstrate that the GPS closely correlates with relapse events and refractory disease in FL patients that mainly received anti-CD20 directed agents. The complexity of individualized risk stratification cannot be reflected adequately by clinical, laboratory or genomic insights alone. The key to optimize personalized predictors of adverse clinical courses will be an integrative analysis of all different aspects to identify high-risk FL patients as early as possible. The GPS could emerge as a contributive determinant in this process. Therefore, the integration of biomarkers of systemic inflammation and decreasing nutritional status combined with cytogenetic as well as molecular genomic signatures in terms of personalized risk stratification in FL patients should be prospectively validated within large-scale randomized trials. In the era of personalized medicine, optimal risk stratification should lead to individual guidance regarding treatment intensity and composition.

## Supplementary Information

Below is the link to the electronic supplementary material.

Supplementary file1 (DOCX 33 kb)

Supplementary file2 (PDF 643 kb)

Supplementary file3 (PDF 186 kb)
