# Supplementary material for: Prognostic impact of nutritional and inflammation-based risk scores in follicular lymphoma in the era of anti-CD20 targeted treatment strategies
Source: J Cancer Res Clin Oncol. 2021 Aug 20;148(7):1789–801. doi: 10.1007/s00432-021-03758-5 (PMC9189087; doi:10.1007/s00432-021-03758-5)
Supplement: Supplementary file 1 — Supplementary file1 (DOCX 33 kb) [file 432_2021_3758_MOESM1_ESM.docx]

**Supplementary Material**

**Supplementary Methods**

The c-index hold discriminatory power of a given statistical model (with higher values indicating superior predictive properties; ≤ 0.5 poor model, no better than predicting an outcome than random chance; 1 = perfect model, impeccable prediction of outcome according to group allocation), while the cAIC quantifies the predictive potential of statistical models upon direct comparison at low sample volumes (with lower values indicating better accuracy). A difference in cAIC values between 0 and 2 indicates the absence of significant differences in model fit while a difference between 2 and 10 suggests an increasing improvement in fit, a difference greater than 10 represents a substantial improvement in fit.

**Treatment modalities**

The majority of FL-patients received a rituximab-based strategy (n =130; 62.2%) and only 32 FL-patients were treated with obinutuzumab (16.7%). With regard to the GPS subgroups, anti-CD20 antibody-based therapy was applied in 93 patients with GPS 0 (70.5%), 37 patients with GPS 1 (90.2%) and 32 patients with GPS 2 (88.9%). The most frequently applied chemotherapeutic backbone was CHOP (cyclophosphamide/ hydroxydaunorubicin/ vincristine/ prednisolone) in 89 cases (42.6%). Bendamustine has been administered in 56 FL-patients as a part of first line treatment. Radiation therapy alone or in combination with pharmacological cytoreductive treatment approaches was initiated in 46 FL-patients (22.0%). Maintenance applying an anti-CD20 targeted therapeutic agent after induction treatment was continued in 90 FL-patients (43.1%). The overall response rate (ORR) after first line therapy was 83.3% while the CR-rate was 39.2% (82/209 cases).

Toxicity profile was mild and predominantly hematological in nature with only 16.3% of patients presented with grade III/IV NCI CTC cytopenia (n = 34). Manifest infections were observed in 29 cases (13.9%).

Second-line therapy in relapse or refractory setting was applied in 89 cases (89/102 patients with r/r FL; 87.3%). In patients with r/r FL, an anti-CD20-antibody based approach was administered in 72 cases (80.9%). Predominantly, FL-patients received bendamustine (n = 35; 39.3%) or a CHOP-like regimen (n = 21; 23.6%) as second line treatment option in relapsed or refractory setting. Reinduction using the same cytoreductive treatment regimen as initially administered in first-line was performed in 11 cases (5.3%) after reasonable responses.

Autologous or allogenic hematopoietic stem cell transplantation (HSCT) were required in 25 cases (11.9%) during the course of the disease. In nine cases (9/25; 36.0%), relapse or refractory disease became manifest within 24 months (POD24).

**Supplementary Figure Legends**

**Supplementary Figure 1.** Comparative age-related survival analysis according to GPS subgroups (**A – D**) consequent upon Pearson’s correlation analysis. Kaplan Meier analysis revealed the GPS to significantly predict OS in both younger (<60 years) and elderly (>60 years) FL-patients (**A, B**). The GPS is a significant predictor of progression-free survival (PFS) in elderly (**D**) but not in younger FL-patients (**C**). Additionally, Pearson’s correlation analysis revealed a close relation between the PNI and sex. Supplementary Figures 1 E – H visualize survival analysis according to a PNI-cutoff value of 50 in females (E, F) and males (G, H).

**Supplementary Figure 2.** Clinical impact of the Follicular Lymphoma International Prognostic Index (FLIPI) in follicular lymphoma patients on both progression-free survival (PFS; A) and overall survival (OS; B). Results from the univariate and the multivariate analysis upon a Cox proportional hazard model could be confirmed within the scope of calculating PFS (A; p = 0.071) and OS (B; p = 0.001) by means of the Kaplan Meier method.

**Supplementary Tables**

**Supplementary Table 1.** 2^nd^ line treatment modalities of all FL-patients.

| **Characteristics** | **Overall study group**  **(n = 102)** | **GPS 0**  **(n = 46)** | **GPS 1**  **(n = 21)** | **GPS 2**  **(n = 35)** |
| --- | --- | --- | --- | --- |
| Best supportive care | 13 (11.9%) | 3 (6.5%) | 4 (19.0%) | 6 (17.1%) |
| **2^nd^ line treatment** | | | | |
| CHOP-like | 21 (23.6%) | 12 (27.9%) | 3 (17.6%) | 6 (20.7%) |
| Bendamustine | 35 (39.3%) | 20 (46.5%) | 8 (47.1%) | 7 (24.1%) |
| R-based | 72 (80.9%) | 38 (88.4%) | 12 (70.6%) | 22 (75.9%) |
| O-based | 6 (6.7%) | 3 (6.9%) | - | 3 (10.3%) |
| Radiation therapy | 8 (8.9%) | 4 (9.3%) | 2 (11.8%) | 2 (6.9%) |
| Auto/Allo Transplant | 14 (15.7%) | 5 (11.6%) | 3 (17.6%) | 6 (20.7%) |
| Other | 11 (12.3%) | 2 (4.7%) | 1 (5.9%) | 8 (27.6%) |
| Anti-CD20 maintenance | 49 (55.1%) | 28 (65.1%) | 7 (41.2%) | 14 (48.3%) |
| **Best response (IWSC)** | | | | |
| CR | 28 (31.5%) | 17 (39.5%) | 6 (35.3%) | 5 (17.2%) |
| PR | 32 (36.0%) | 16 (37.2%) | 9 (52.9%) | 7 (24.1%) |
| SD | 15 (16.8%) | 9 (20.9%) | 1 (5.9%) | 5 (17.2%) |
| PD | 14 (15.7%) | 1 (2.3%) | 1 (5.9%) | 12 (41.4%) |
| **Toxicity profile (NCI CTC)** | | | | |
| Cytopenia grad III/IV | 19 (21.3%) | 6 (13.9%) | 4 (23.5%) | 9 (31.0%) |
| Neutropenia related fever | 6 (6.7%) | 2 (4.7%) | 2 (11.8%) | 2 (6.9%) |
| Pneumonia | 2 (2.2%) | 2 (4.7%) | - | - |
| Sepsis | 3 (3.4%) | 1 (2.3%) | 1 (5.9%) | 1 (3.4%) |
| Neuropathy | 3 (3.4%) | 2 (4.7%) | - | 1 (3.4%) |
| Cardiotoxicity | 2 (2.2%) | - | - | 2 (6.9%) |
| Mucositis | 9 (10.1%) | 5 (11.6%) | 2 (11.8%) | 2 (6.9%) |
| CHOP, cyclophosphamide/hydroxydaunorubicin/vincristine/prednisolone; CR, complete remission; GPS, Glasgow-prognostic score; HSCT, hematopoietic stem cell transplantation; IWSC, International Workshop criteria; NCI CTC, National Cancer Institute Common Toxicity Criteria; O, obinutuzumab; PD, progressive disease; POD24, progression of disease within 24 months; PR, partial remission; R, rituximab; SD, stable disease. | | | | |

**Supplementary Table 2.** 3^rd^ line treatment modalities of all FL-patients.

| **Characteristics** | **Overall study group**  **(n = 46)** | **GPS 0**  **(n = 20)** | **GPS 1**  **(n = 8)** | **GPS 2**  **(n = 18)** |
| --- | --- | --- | --- | --- |
| Best supportive care | 6 (13.0%) | 1 (5.0%) | 1 (12.5%) | 4 (22.2%) |
| **3^rd^ line treatment** | | | | |
| CHOP-like | 4 (10.0%) | 2 (10.5%) | 1 (14.3%) | 1 (7.1%) |
| Bendamustine | 14 (35.0%) | 6 (31.6%) | 1 (14.3%) | 7 (50.0%) |
| R-based | 20 (50.0%) | 13 (68.4%) | 4 (57.1%) | 3 (21.4%) |
| O-based | 3 (7.5%) | 2 (10.5%) | - | 1 (7.1%) |
| Radiation therapy | 3 (7.5%) | - | 1 (14.3%) | 2 (14.3%) |
| Auto/Allo Transplant | 11 (27.5%) | 9 (47.4%) | 1 (14.3%) | 1 (7.1%) |
| Other | 8 (20.0%) | 2 (10.5%) | 3 (42.9%) | 3 (21.4%) |
| Anti-CD20 maintenance | 14 (35.0%) | 9 (47.4%) | 2 (28.6%) | 3 (21.4%) |
| **Best response (IWSC)** | | | | |
| CR | 12 (30.0%) | 11 (57.9%) | 1 (14.3%) | - |
| PR | 10 (25.0%) | 3 (15.8%) | 2 (28.6%) | 5 (35.7%) |
| SD | 6 (15.0%) | 3 (15.8%) | 1 (14.3%) | 2 (14.3%) |
| PD | 12 (30.0%) | 2 (10.5%) | 3 (42.9%) | 7 (50.0%) |
| **Toxicity profile (NCI CTC)** | | | | |
| Cytopenia grad III/IV | 12 (30.0%) | 6 (31.6%) | 3 (42.9%) | 3 (21.4%) |
| Neutropenia related fever | 3 (7.5%) | 2 (10.5%) | 1 (14.3%) | - |
| Pneumonia | - | - | - |  |
| Sepsis | 2 (5.0%) | 2 (10.5%) | - | - |
| Neuropathy | 1 (2.5%) | - | - | 1 (7.1%) |
| Cardiotoxicity | 1 (2.5%) | 1 (5.2%) | - | - |
| Mucositis | 5 (12.5%) | 4 (21.1%) | - | 1 (7.1%) |
| CHOP, cyclophosphamide/hydroxydaunorubicin/vincristine/prednisolone; CR, complete remission; GPS, Glasgow-prognostic score; HSCT, hematopoietic stem cell transplantation; IWSC, International Workshop criteria; NCI CTC, National Cancer Institute Common Toxicity Criteria; O, obinutuzumab; PD, progressive disease; POD24, progression of disease within 24 months; PR, partial remission; R, rituximab; SD, stable disease. | | | | |

**Supplementary Table 3.** Data on model fit and concordance of CRP and albumin-based risk scores in FL patients.

|  | **OS** | | **PFS** | |
| --- | --- | --- | --- | --- |
| **Model** | **c-index** | **cAIC** | **c-index** | **cAIC** |
| **GPS** | 0.761 | 2326 | 0.595 | 2207 |
| **CAR** | 0.709 | 2329 | 0.575 | 2211 |
| cAIC, corrected Akaike’s information criterion; CAR, CRP/albumin ratio; CI, confidence interval; GPS, Glasgow prognostic score. | | | | |

Both the GPS as well as CAR were shown to hold predictive properties for both OS and PFS while the GPS was shown to be superior compared to CAR. We calculated a higher c-index for the GPS and a lower value for the cAIC of GPS indicating preponderance regarding survival prediction (cAIC differences for OS: 3 and PFS: 4).

**Supplementary Table 4.** Progression-free and overall survival in univariate analysis (Univariate cox analysis) after exclusion of FL-patients suffering from cachexia or obesity.

| **Univariate analysis** | | | | |
| --- | --- | --- | --- | --- |
|  | **PFS** | | **OS** | |
| **Prognostic factor** | **p-value** | **HR (95% CI)** | **p-value** | **HR (95% CI)** |
| GPS | **<0.0001** | 1.608 (1.254 – 2.063) | **<0.0001** | 5.935 (3.487 – 10.10) |
| CRP | **0.001** | 2.189 (1.404 – 3.412) | **<0.0001** | 12.75 (5.516 – 29.47) |
| Albumin | **0.001** | 2.107 (1.354 – 3.279) | **<0.0001** | 35.29 (10.70 – 116.4) |
| NLR | 0.605 | 0.934 (0.723 – 1.208) | 0.075 | 1.440 (0.964 – 2.150) |
| PI  PNI | **0.003** | 1.679 (1.197 – 2.356) | **<0.0001** | 4.677 (2.752 – 7.949) |
|  | 0.860 | 0.962 (0.626 – 1.478) | **0.001** | 0.219 (0.090 – 0.533) |
| Age > 60 years | 0.068 | 0.472 (0.211 – 1.057) | 0.323 | 0.574 (0.191 – 1.727) |
| B-Symptoms | 0.125 | 1.495 (0.895 – 2.497) | 0.067 | 2.003 (0.952 – 4.213) |
| ECOG PS ≥ 2 | 0.066 | 1.742 (0.963 – 3.152) | **<0.0001** | 6.719 (3.060 – 14.75) |
| Elevated LDH | **0.038** | 1.635 (1.028 – 2.600) | **<0.0001** | 6.313 (3.113 – 12.80) |
| BM involvement | **0.004** | 1.892 (1.222 – 2.928) | 0.150 | 1.662 (0.832 – 3.320) |
| CCI > 3 | 0.390 | 0.827 (0.537 – 1.275) | **0.001** | 4.196 (1.738 – 10.13) |
| FLIPI | 0.184 | 1.146 (0.937 – 1.400) | **0.001** | 1.653 (1.229 – 2.222) |
| Ann Arbor | **0.002** | 1.535 (1.175 – 2.007) | 0.054 | 1.562 (0.992 – 2.460) |
| CAR, C-reactive-protein-albumin ratio; CCI, Charlson Comorbidity Index; CRP, C-reactive protein; ECOG PS, Eastern Cooperative Oncology Group performance status; FLIPI, Follicular Lymphoma International Prognostic Index; GPS, Glasgow Prognostic Score; HR, Hazard ratio; LDH, lactate dehydrogenase; NLR, neutrophil-to-lymphocyte ratio; OS, overall survival; PFS, progression-free survival; PI, prognostic index.  Bold values indicate statistical significance (P < 0·05) in univariate cox analysis. | | | | |

**Supplementary Table 5.** Overall survival and progression free survival in univariate analysis and consecutive multivariate Cox proportional hazard regression after exclusion of FL-patients suffering from cachexia or obesity.

|  | **Univariate analysis OS** | **Multivariate analysis OS** | |
| --- | --- | --- | --- |
| **Prognostic factor** | p-value | p- value | HR (95% CI) |
| GPS | **<0.0001** | **<0.0001** | 5.572 (2.474 – 12.549) |
| PI* | **<0.0001** | 0.538 | 0.689 (0.210 – 2.258) |
| PNI* | **0.001** | 0.531 | 0.727 (0.269 – 1.970) |
| CCI>3 | **0.001** | 0.085 | 2.366 (0.888 – 6.304) |
| FLIPI | **0.001** | **0.049** | 1.380 (1.001 – 1.903) |
| LDH | **<0.0001** | **0.007** | 3.174 (1.377 – 7.313) |
|  | | | |
|  | **Univariate analysis PFS** | **Multivariate analysis PFS** | |
|  | p-value | p-value | HR (95% CI) |
| GPS | **<0.0001** | **0.009** | 1.737 (1.147 – 2.631) |
| PI* | **0.003** | 0.990 | 0.996 (0.574 – 1.728) |
| PNI* | 0.860 | 0.176 | 1.394 (0.862 – 2.256) |
| CCI>3 | 0.390 | 0.082 | 0.655 (0.406 – 1.056) |
| FLIPI | 0.184 | 0.195 | 1.160 (0.927 – 1.452) |
| LDH | **0.038** | 0.787 | 1.075 (0.635 – 1.822) |
| CCI, Charlson Comorbidity Index; FLIPI, Follicular Lymphoma International Prognostic Index; GPS, Glasgow Prognostic Score; OS, overall survival; PFS, progression free survival; PI, Prognostic Index; PNI, Prognostic Nutritional Index.  * CRP > 10mg/dl, white blood cell count >11.000/μl  ** > 50 | | | |
