# Supplementary figures and images for: Prognostic impact of nutritional and inflammation-based risk scores in follicular lymphoma in the era of anti-CD20 targeted treatment strategies
Source: J Cancer Res Clin Oncol. 2021 Aug 20;148(7):1789–801. doi: 10.1007/s00432-021-03758-5 (PMC9189087; doi:10.1007/s00432-021-03758-5)

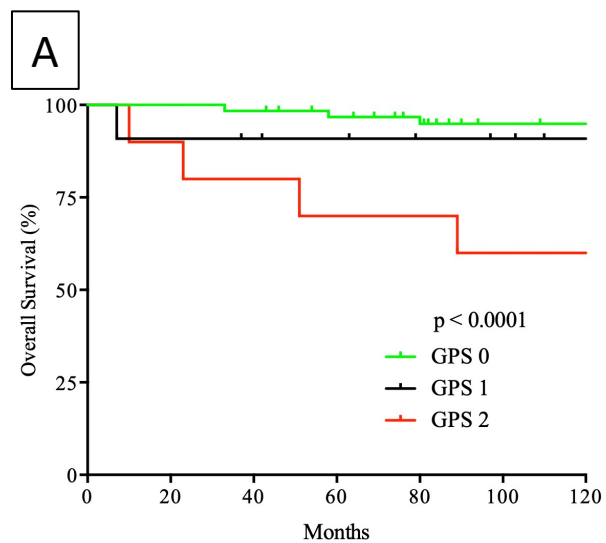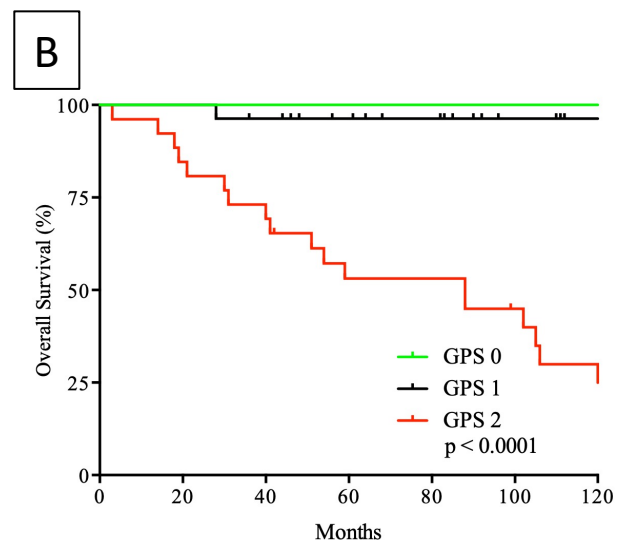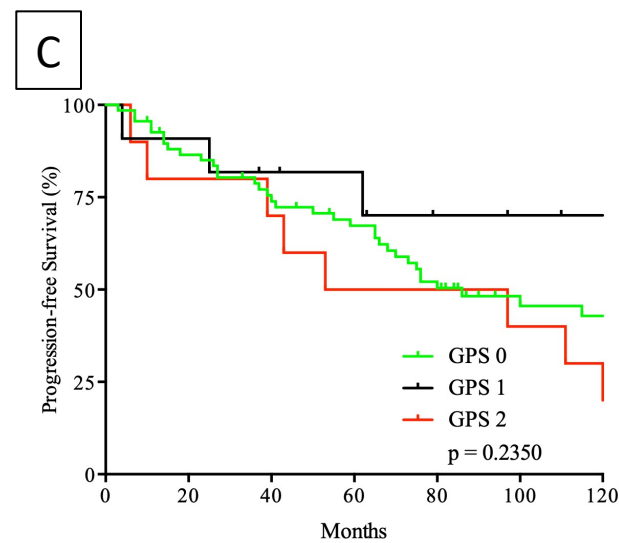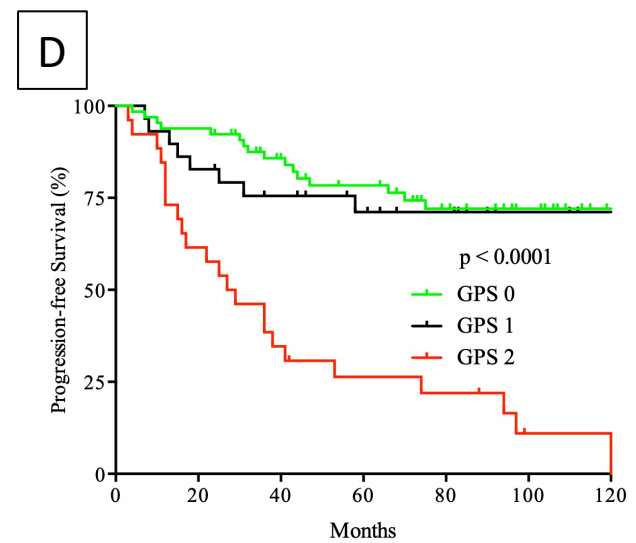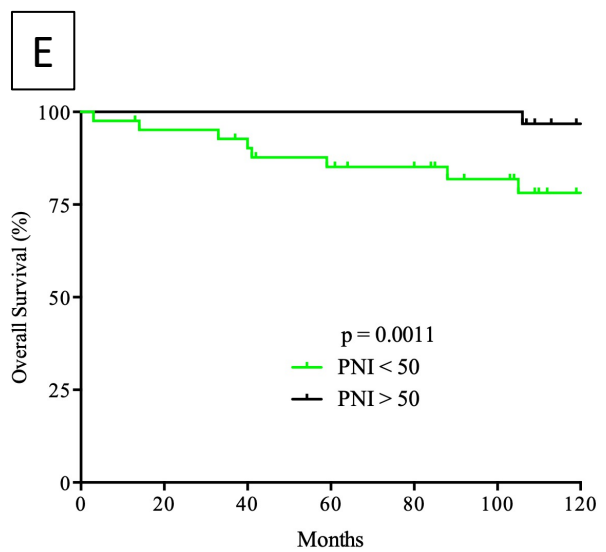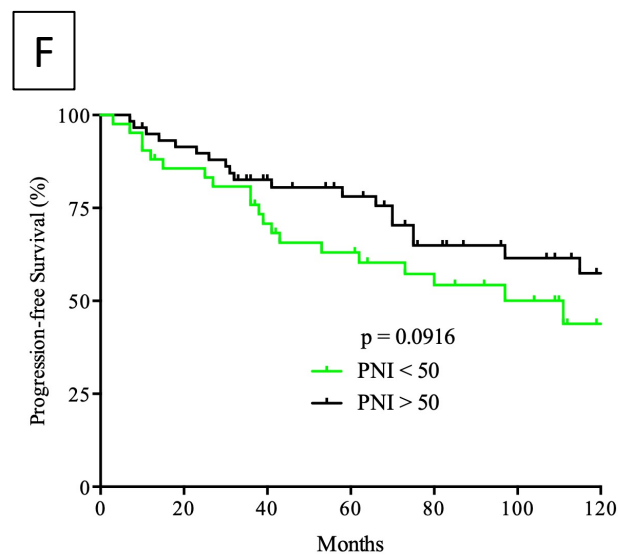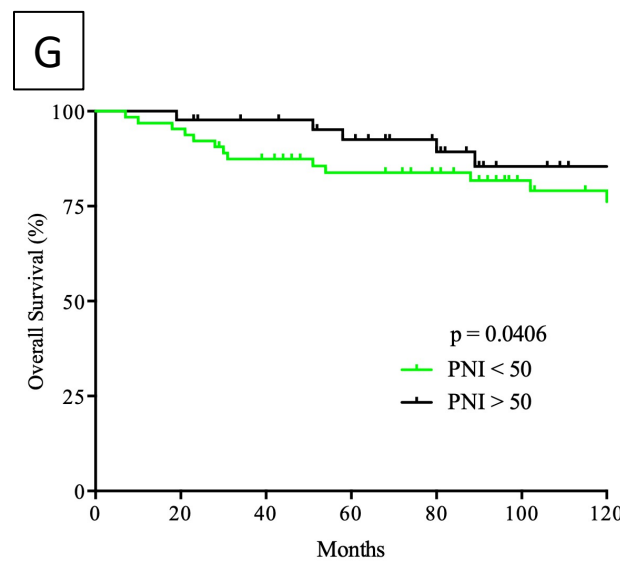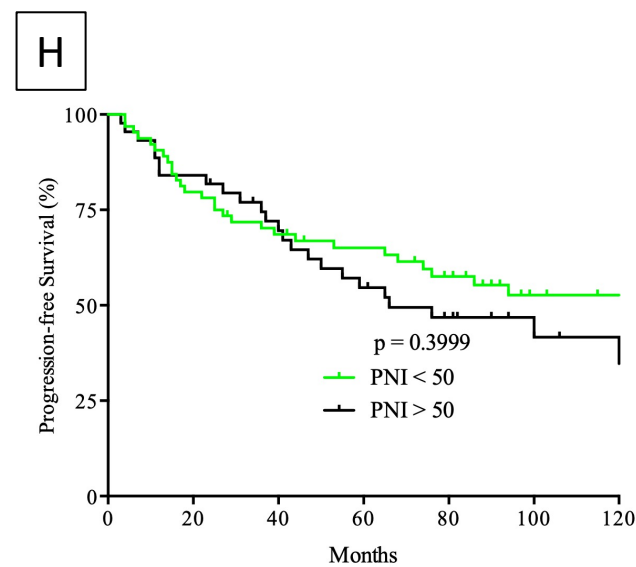

Supplement: Supplementary file 2 — Supplementary file2 (PDF 643 kb) [file 432_2021_3758_MOESM2_ESM.pdf]

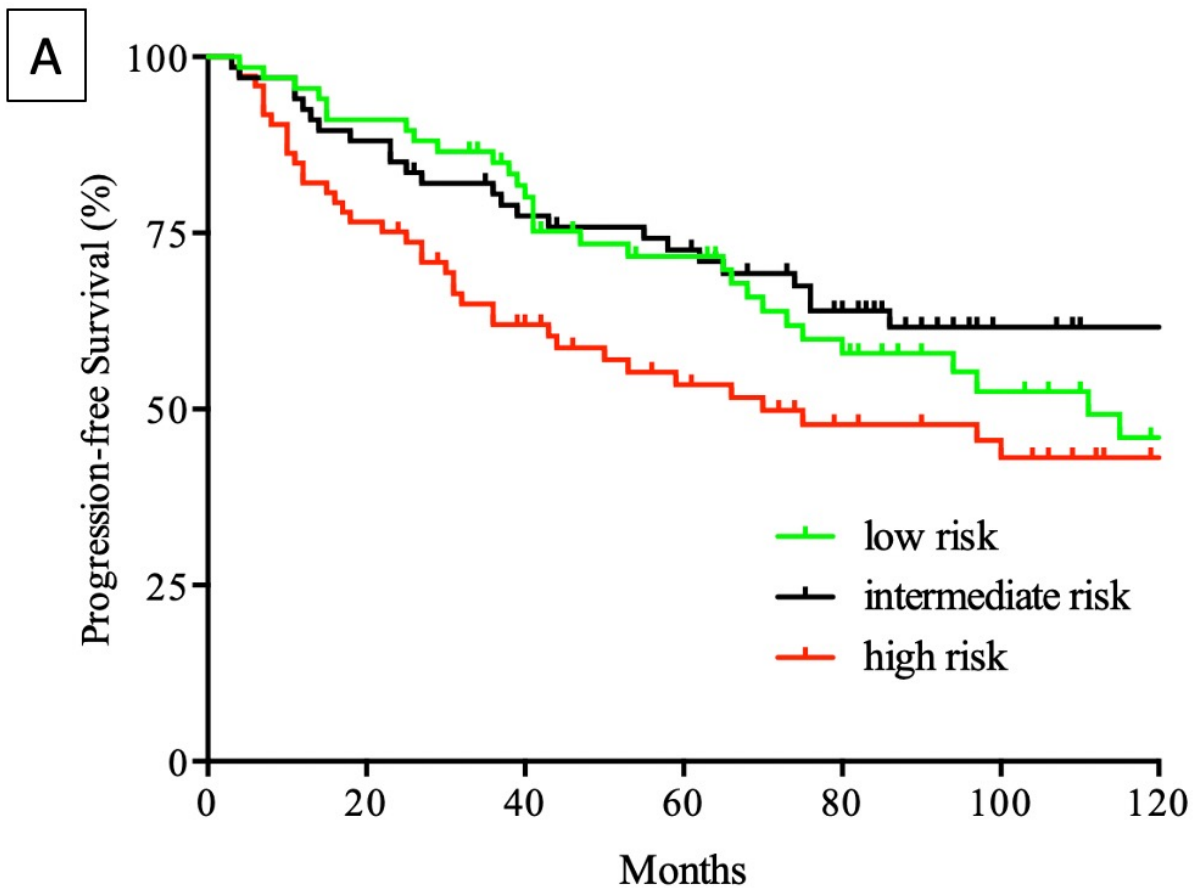

|    |    |    |    |    |    |    |
|----|----|----|----|----|----|----|
| 67 | 63 | 50 | 41 | 30 | 20 | 13 |
| 67 | 60 | 51 | 46 | 35 | 18 | 15 |
| 73 | 56 | 40 | 31 | 24 | 19 | 13 |

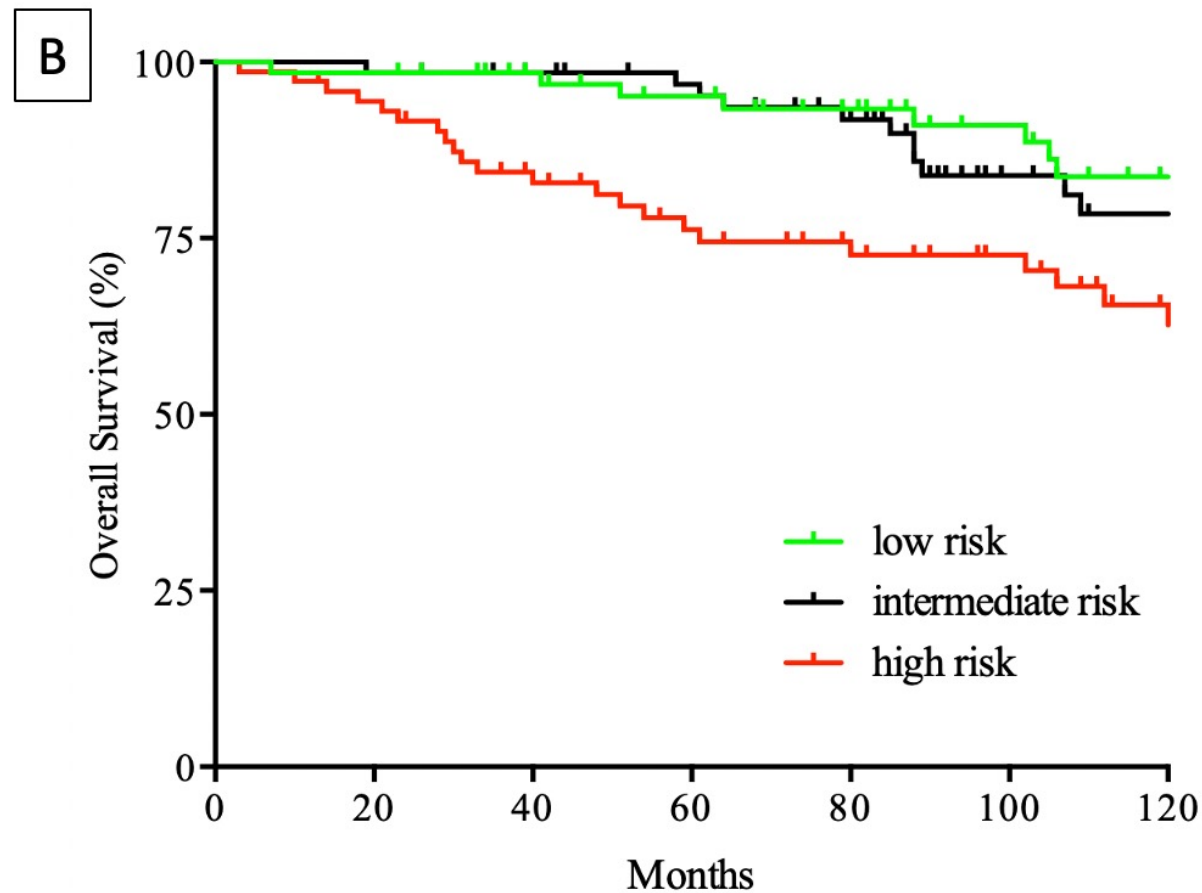

|    |    |    |    |    |    |    |
|----|----|----|----|----|----|----|
| 67 | 67 | 61 | 55 | 48 | 39 | 32 |
| 67 | 67 | 64 | 60 | 52 | 33 | 29 |
| 73 | 68 | 56 | 46 | 39 | 34 | 24 |

Supplement: Supplementary file 3 — Supplementary file3 (PDF 186 kb) [file 432_2021_3758_MOESM3_ESM.pdf]
